# Effect of dietary supplementation with *Allium mongolicum* Regel extracts on growth performance, carcass characteristics, and the fat color and flavor-related branched-chain fatty acids concentration in ram lambs

**DOI:** 10.5713/ajas.20.0246

**Published:** 2020-10-14

**Authors:** Wangjing Liu, Changjin Ao

**Affiliations:** 1 College of Animal Science and Technology, Gansu Agricultural University, Lanzhou, Gansu 730070, China; 2 Inner Mongolia Key Laboratory of Animal Nutrition and Feed Science, College of Animal Science, Inner Mongolia Agricultural University, Hohhot Inner Mongolia 010018, China

**Keywords:** *Allium mongolicum* Regel, Branched-chain Fatty Acids, Carcass Characteristics, Color, Growth Performance

## Abstract

**Objective:**

This study aimed to investigate the effect of dietary supplementation with *Allium mongolicum* Regel extracts on the growth performance, carcass characteristics, fat color, and concentrations of three branched-chain fatty acids related to flavor in ram lambs.

**Methods:**

Sixty 3-month-old, male, small-tailed Han sheep were selected and randomly allocated into four groups in a randomized block design. Four feeding treatments were used: i) a basal diet without supplementation as the control group (CK); ii) the basal diet supplemented with 10 g/lamb/d *Allium mongolicum* Regel powder as the AMR group; iii) the basal diet supplemented with 3.4 g/lamb/d *Allium mongolicum* Regel water extract as the AWE group; and iv) the basal diet supplemented with 2.8 g/lamb/d *Allium mongolicum* Regel ethanol extract as the AFE group.

**Results:**

The results demonstrated that the dry matter intake was lower for the AFE group than that in other groups (p = 0.001). The feed conversion ratio was greater for the AFE than that in other groups (p = 0.039). Dietary supplementation with *Allium mongolicum* Regel powder and its extracts decreased the concentrations of 4-methyloctanoic acid (MOA) (p<0.001), 4-ethyloctanoic acid (EOA) (p<0.001), and 4-methylnonanoic acid (MNA) (p = 0.044) in perirenal adipose tissue compared to those observed in the CK lambs. Dietary supplementation with *Allium mongolicum* Regel powder and its extracts decreased the concentrations of MOA (p<0.001) and EOA (p<0.001) in dorsal subcutaneous adipose tissue compared to those in the CK lambs. The concentrations of MOA (p<0.001) and EOA (p = 0.002) in omental adipose tissue were significantly affected by treatment, although there was a tendency for lower MNA (p = 0.062) in AMR, AWE, and AFE lambs than that in CK lambs.

**Conclusion:**

This study demonstrated that *Allium mongolicum* Regel and its extracts could significantly promote feed efficiency, although dry matter intake decreased and could decrease the MOA and EOA concentrations related to characteristic flavor and odor of body fat in lambs, except for tail adipose tissue.

## INTRODUCTION

For several decades the daily weight gain, feed efficiency, and carcass composition of lambs were greatly improved by adding growth promoters to their feed. Human health problems caused by traditional growth promoters (i.e., antibiotic growth promoters) in animal feed were the main reason for many consumers rejection. At present, many countries have announced the prohibition of traditional feed additives and instead are searching for alternatives [1]. In this context, plant extracts have emerged as alternative growth promoters that can be used in the lamb finishing stages while being accepted by consumers [2]. On the one hand, there are a variety of compounds with different biological activities from plant extracts that can affect the growth performance of animals [3]. Lambs fed grapefruit peel extracts showed an increased average daily weight gain (ADG) [4]. However, several studies have reported that due to their strong odor and flavor dietary supplementation with plant extracts can decrease the average daily feed intake of lambs [5]. On the other hand, many studies have reported that natural plants and their extracts affect meat color and firmness. Jiang et al [6] reported that dietary tomato pomace decreased meat yellowness and chroma. Dietary pomegranate by-product silage improved fat color and firmness, which might have been the result of enrichment of the meat with phenolic compounds [7].

Branched-chain fatty acids (BCFAs), such as 4-methy loctanoic acid (MOA), 4-ethyloctanoic acid (EOA), and 4-methylnonanoic acid (MNA), are the compounds thought to be responsible for the flavor and odor of sheep meat and are deposited in the fat by the animal, moreover, their metabolites are released into the bloodstream [8]. Ameliorating the impacts of BCFAs on the sensory components of meat would result in higher acceptance of the final product by consumers [9]. At present, there are many studies about the effect of plant extracts on BCFAs concentrations in sheep fat, but the results are not consistent [10]. The possible reason is that the levels of nutrients forage grass vary between seasons [11]. Feeding with quebracho tannins, a powder extract made from tree bark [12], and grape seed extract [13] was shown to reduce the production of 3-methylindole in sheep fed concentrate feed, which influences the acceptance of consumption related to the flavor of this chemical in sheep meat. Therefore, feed or feed additives used as feed supplements can affect sheep meat odor and flavor. Many studies have reported that feed has a strong impact on the concentrations of BCFAs in lamb tissue. However, limited references are found in the literature regarding the effect of plant extracts on the concentrations of BCFAs related to flavor.

A type of perennial and xerophytic *Liliaceous allium* plant, *Allium mongolicum* Regel (Mongolian leek), grows in desertified grassland regions in northern China and is considered as one specific food source that produces a pleasant meat flavor of mutton. *Allium mongolicum* Regel has a unique flavor, and is rich in protein, flavonoids, polysaccharide, and other components [14]. According to the Mongolian medicine records, *Allium mongolicum* Regel has various special effects such as lowering blood pressure, hypolipidemic, stimulating the appetite, and replenishing kidneys. A recent study reported that dietary polysaccharides from *Allium mongolicum* Regel may increase the weight gain, specific growth rate, and feed conversion ratio (FCR) of *Channa argus* [14]. However, few studies have focused on the application of *Allium mongolicum* Regel and its extracts in ruminants. Our previous study also showed that the addition of flavonoids from *Allium mongolicum* Regel to the diet improved sheep meat quality and had a positive effect on the reduction of characteristic flavor and odor of *longissimus dorsi* muscle in lambs [15]. To our knowledge, no information on the BCFAs concentrations of different types of adipose tissue from small-tailed Han sheep is available in the scientific literature. Moreover, previous studies have reported that the *Allium mongolicum* Regel water extract and ethanol extract have higher phenolic contents and flavonoid contents [16]. We hypothesized that these *Allium mongolicum* Regel extracts could improve the growth performance, carcass composition, and fat color and reduce the level of BCFAs in adipose tissue. The objectives of this study were to evaluate the effects of *Allium mongolicum* Regel and its extracts on growth performance, carcass characteristics, fat color, and the concentrations of three BCFAs related to the characteristic flavor and odor in ram lambs.

## MATERIALS AND METHODS

### Animal care

This study was carried out following the recommendations of the Instructive Notions concerning Caring for Experimental Animals, Ministry of Technology of China. All experimental procedures involving animals were evaluated and approved by the guidelines of the Animal Care and Use Committee of Inner Mongolia Agriculture University (Hohhot, China).

### Extraction process of the *Allium mongolicum* Regel water extract

Dried powder of *Allium mongolicum* Regel leaves was purchased from Hao Hai Biological Company (Alxa League, Inner Mongolia, China). *Allium mongolicum* Regel water and ethanol extracts were provided by the Animal Nutrition and Immunology Laboratory of Inner Mongolia Agricultural University. Briefly, *Allium mongolicum* Regel powder was added to distilled water at a ratio of 1:20 (w/v) and placed in a water bath (HH-8, Guohua Electric Appliance Co., Ltd., Jiangsu, China) set at 80°C for 8 h. Then, the mixture was filtered with a vacuum filter system (SHB-III, Zhengzhou Greatwall Scientific Industrial and Trade Co., Ltd., Henan, China) to obtain the filtrate. After that, the filtrate was concentrated with a rotary evaporator (IKA HB10, IKA India Private Limited, Staufen, Germany) at 78°C and a speed of 60 rpm (model: R1002B, Yamato Scientific, Beijing, China), lyophilized in a freeze-dryer (CA301, Yamato Scientific, China) to remove any remaining water and then pulverized.

### Extraction process of *Allium mongolicum* Regel ethanol extract

Seventy-five percent ethanol (80176961, Sinopharm Chemical Reagent Co., Ltd., Shanghai, China) was mixed with *Allium mongolicum* Regel powder at a ratio of 5:1 (v/w), and the mixture was sonicated in an ultrasonic bath (KQ-300DE, Kunshan Ultrasonic Instruments Co., Ltd., Jiangsu, China) (75 watts, 15 min). Then, the ethyl alcohol was evaporated from the mixture by a rotary evaporator (IKA HB10, IKA India Private Limited, Germany). The concentrate was lyophilized in a freeze-dryer (CA301, Yamato Scientific, China) and then pulverized. Previous studies reported that the *Allium mongolicum* Regel water extract and ethanol extract, having higher phenolic contents (10.28 and 8.50 mg/g dry weight, respectively) and flavonoid contents (4.52 and 3.92 mg/g dry weight, respectively) [16].

### Diet and feeding

The sheep feeding trial was conducted at the Fuchuan company, located in Bayannaoer city, Inner Mongolia Autonomous Region, China (40°79′N, 107 42′E, 1,038 m above sea level). Sixty 3-month-old, male, small-tailed Han sheep (average body weight, 23.67±3.43 kg) were selected and randomly allocated into four groups in a randomized block design, with each group composed of three units, five lambs in each unit. Four dietary treatments were used: i) a basal diet without supplementation as the control group (CK, n = 15); ii) the basal diet supplemented with 10 g/lamb/d *Allium mongolicum* Regel powder as the AMR group (AMR, n = 15); iii) the basal diet supplemented with 3.4 g/lamb/d *Allium mongolicum* Regel water extract as the AWE group (AWE, n = 15); and v) the basal diet supplemented with 2.8 g/lamb/d *Allium mongolicum* Regel ethanol extract as the AFE group (AFE, n = 15). One hundred grams concentrate of the basal diet was mixed with 10 g *Allium mongolicum* Regel powder, 3.4 g water extract, or 2.8 g ethanol extract, respectively, and divided each mixture into two equal amounts at 0800 h and 1730 h, then they were provided for individual sheep twice daily to ensure that the supplements were completely consumed by each lamb. The dose of *Allium mongolicum* Regel (10 g/lamb/d) was based on preliminary field and research studies [17]. Three feeding treatments (AMR, AWE, and AFE groups) consumed the same amount of *Allium mongolicum* Regel powder. The doses of water extract (3.4 g/lamb/d) and ethanol extract (2.8 g/lamb/d) in the diet were calculated according to their extraction rates (34% and 28%, respectively) from the *Allium mongolicum* Regel powder.

The lambs were given free access to drinking water. The experiment lasted for 75 days, including a 15-day preliminary feeding period for adaptation and a 60-day experimental feeding period. Before the beginning of the experiment, all animals were treated for internal and external parasites and given routine vaccinations. Respective treatment’s diets were offered to the animals twice daily at 0700 h and 1800 h, and the body weights were measured on the first and last days of the experimental feeding period before the morning meal. The ADG for individual sheep was calculated using the sum of the ADGs determined during the experimental period divided by the number of days during the experimental period.

### Sample collection

Feed intake was recorded daily for the groups of 5 lambs based on the amount of feed offered and refusals to determine voluntary dry matter intake (DMI). Samples of diet were collected in separate self-sealing bags and stored at −20°C for chemical analysis. At the end of the feeding experiment, two lambs from each unit were harvested after an 18-hour fast by standard Halal procedures according to Santos et al [18]. The transport distance to the slaughterhouse (Inner Mongolia Little Sheer Meat Industry Co., LTD., Bayannaoer, China) was approximately 14 km. The time waiting before slaughter was less than 3 h. Lambs were weighed before loading. Eviscerated carcasses were then weighed to determine the hot carcass weight (HCW) and chilled at 4°C±1°C in the dark. The carcass yield was calculated as the percentage of the HCW per body weight at slaughter. The perirenal adipose tissue (PF) weight and dorsal subcutaneous adipose tissue (DF) thickness over the last thoracic rib and firmness (finger test on a scale from 3 [oily] to 15 [very hard]) were measured after slaughter. Subsequently, the PF, DF, tail adipose tissue (TF), and omental adipose tissue (OF) samples were wrapped in aluminum foil, vacuum-packed in sealable polyamide bags, flash-frozen in liquid N2 and stored at −80°C until BCFAs analysis. All samples weighed at least 30 g.

### Chemical analysis

#### Analysis of feed

The total mixed rations for each treatment were analyzed for dry matter (Method 930.15), nitrogen (Method 984.13), and ether extract (Method 920.39) content according to the AOAC [19], and crude protein was calculated as 6.25× the nitrogen content. For individual mineral (Ca and P) analyses, the samples were analyzed with a Thermo Jarrell Ash IRIS Advantage HX inductively coupled plasma radial spectrometer (Thermo Instrument System, Inc., Waltham, MA, USA). Neutral detergent fiber (NDF) and acid detergent fiber were measured according to the method of Van Soest [20] with an ANKOM 200 fiber analyzer (A200, ANKOM Technology Corporation, New York, USA) and fiber filter bags (F57, ANKOM Technology Corporation, USA). Heat stable amylase was not used in the NDF determination, and the results are shown in Table 1.

#### Color of fat

The colors of PF, TF, DF, and OF were measured at 15°C to 22°C ambient temperature using an OPTO-LAB MATTHAUS (Matthaus GmbH & Co. KG, Berlin, Germany). Color coordinates were expressed as lightness (L*), redness (a*), yellowness (b*), chroma (C*), and hue angle (H*) [21]. Values are expressed as the mean of three different measurements per fat sample.

#### Measurement of BCFAs

Volatile 4-alkyl-branched fatty acids are characteristic flavor compounds of sheep and goat. Methyl or ethyl substitution occurred on even-numbered carbon atoms (relative to the carboxyl group) and the chain lengths of the acids ranged from 9 to 10 carbon atoms [9]. The MOA, MNA, EOA, and undecanoic acids (the last of which was used as the internal standard) with a purity of >98% were purchased from Sigma Aldrich (Milwaukee, WI, USA). The measurement of BCFAs was carried out as described by Watkins et al [22]. The surface lays of fat samples were removed, and remainder cut into 0.5 cm3 squares approximately. Molten fat was prepared by heating the cut portions in a microwave oven for approximately 5 mins, ensuring homogeneity of the sample. The fatty acid-trimethyl esters were separated by injection (1 μL) onto a DB5-MS fused silica capillary column (J&W, 30 m×0.25 mm i.d. ×250 μm film thickness) in a Varian 3400 gas chromatograph (GC) and detected by a Saturn 2000 ion trap mass spectrometer operating in full scan mode. Quantitation of the BCFAs was performed using the Varian Saturn Workstation 2000 software.

### Statistical analysis

One-way analysis of variance was used to test the effects of dietary treatments on growth performance, carcass composition, and the concentrations of BCFAs of body fat in each treatment by SAS (v 9.0) for 4 treatments (SAS Inst. Inc., Cary, NC, USA) [23]. The experimental unit was individual animal. Differences among means were tested using Duncan multiple range tests [24]. The results are presented as the mean values and standard error of the mean. The data means were considered significantly different at p-value<0.05, and tendencies were considered at 0.05<p-value<0.10. Boxplots of the concentration of MOA (n = 24), EOA (n = 24), and MNA (n = 24) in different adipose tissues of lambs were visualized using the GraphPad Prism 5 package. The upper and lower whiskers represent the extreme values, and the line in the box indicates the median value. Spearman’s rank correlation coefficients analysis (ρ) among the three BCFAs (MOA, MNA, and EOA) in different adipose tissues was analyzed via the corr. test of the psych package in R software (v 2.15.3) (n = 24).

## RESULTS

### Animal growth performance and carcass characteristics

The animal growth performance and carcass characteristics did not differ (p>0.05) between feeding treatments, except for DMI and FCR (Table 2). The DMI was lower for the AFE than for the other groups (p = 0.001). The DMI was 1.83 kg for the AFE and increased to 1.99 kg for the AWE, 1.99 kg for the AMR, and 1.96 kg for the CK. Moreover, there was a greater FCR in the AFE than in the other groups (p = 0.039), but the FCR was not significantly different among the CK, AMR, and AWE lambs. The ADG during the experiment, HCW and CY averaged 245 g/d, 21.3 kg, and 52.8%, respectively. The dorsal subcutaneous fat thickness and perirenal fat weight averaged 3.0 mm and 222 g, respectively.

### Color of fat

The TF, PF and DF L*, a*, b*, C*, and H* values were not affected (p>0.050) by treatment, although there was a tendency (p = 0.096) for a lower b* value of the PF in AMR lambs than in AFE lambs (Table 3). There were no treatment effects on the color coordinates of the OF, except for a* (p = 0.044), which was greater for AFE lambs than for AMR and AWE lambs. Besides, compared with that of the CK, AWE, and AFE lambs, AMR lambs had decreased values of C* (p = 0.021) in the OF. Moreover, the value of b* tended (p = 0.087) to be lower for the OF in AMR lambs than in AFE lambs, but values for the AFE lambs did not differ (p> 0.050) from those of either CK or AWE lambs.

### Effects of supplementation with *Allium mongolicum* Regel and its extracts on three BCFAs

Dietary supplementation with *Allium mongolicum* Regel and its extracts decreased the concentrations of MOA (p<0.001), EOA (p<0.001), and MNA (p = 0.044) in the PF compared to those in the PF of the CK (Table 4). The concentrations of MOA were lower in the PF (p<0.050) for AFE lambs than for AMR and AWE lambs. Dietary supplementation with *Allium mongolicum* Regel and its extracts decreased the concentrations of MOA (p<0.001) and EOA (p<0.001) in the DF compared to those in the DF of the CK. The concentrations of MOA (p<0.001) and EOA (p = 0.002) in the OF were affected by treatment, although there was a tendency for lower (p = 0.062) MNA concentrations in the OF of AMR, AWE, and AFE lambs than in the OF of CK lambs. Moreover, MOA was the most abundant of the BCFAs, followed by EOA and MNA in all types of adipose tissue.

### Concentrations of three BCFAs in different adipose tissues

**Figure 1:**
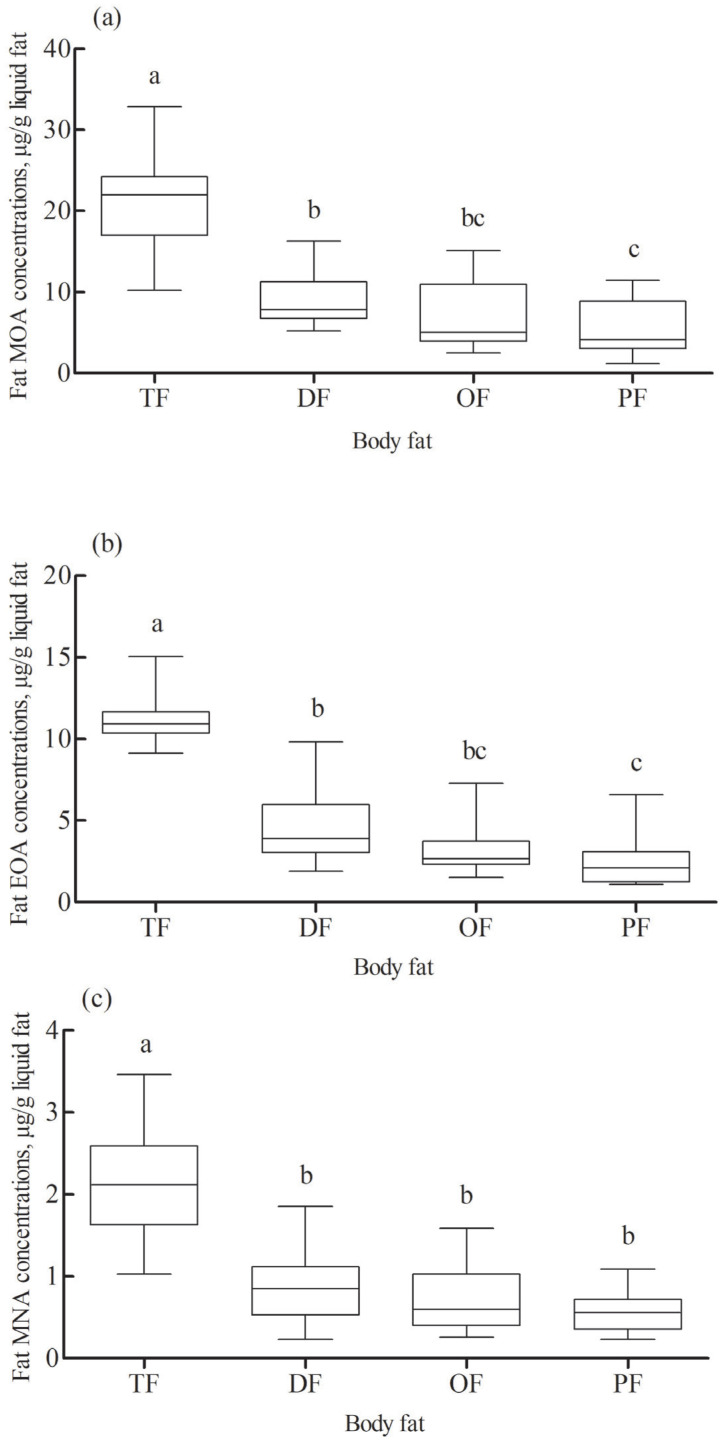
Boxplots of the concentration of MOA (a, n = 24), EOA (b, n = 24), and MNA (c, n = 24) in the tail adipose tissue (TF), dorsal subcutaneous adipose tissue (DF), omental adipose tissue (OF), and perirenal adipose tissue (PF) in lambs. The upper and lower whiskers represent the extreme values, and the line in the box indicates the median value. ^a–c^ Mean values with different letters are different at p<0.05. MOA, 4-methyloctanoic acid; EOA, 4-ethyloctanoic acid; MNA, 4-methylnonanoic acid.

The concentrations of three BCFAs in different types of adipose tissues are shown in Figure 1. The concentrations of MOA (Figure 1a), EOA (Figure 1b), and MNA (Figure 1c) were greater (p<0.001) in the TF than in the DF, OF, and PF. Both the concentrations of MOA and EOA were lower (p< 0.001) in the PF than in the DF. The concentrations of MOA averaged 20.99 μg/g in the TF (range: 10.21 to 32.87 μg/g), 9.26 μg/g in the DF (range: 5.24 to 16.27 μg/g), 6.80 μg/g in the OF (range: 2.46 to 15.11 μg/g) and 5.21 μg/g in the PF (range: 1.17 to 11.43 μg/g). The concentrations of EOA averaged 11.04 μg/g in the TF (range: 9.12 to 15.04 μg/g), 4.84 μg/g in the DF (range: 1.89 to 9.46 μg/g), 3.59 μg/g in the OF (range: 1.52 to 7.28 μg/g) and 2.60 μg/g in the PF (range: 1.10 to 6.59 μg/g). The concentrations of MNA averaged 2.11 μg/g in the TF (range: 1.10 to 3.46 μg/g), 0.92 μg/g in the DF (range: 0.23 to 1.85 μg/g), 0.74 μg/g in the OF (range: 0.26 to 1.58 μg/g) and 0.61 μg/g in the PF (range: 0.26 to 1.09 μg/g).

### Correlation analysis of three BCFAs in different adipose tissues

Correlation analysis of the three BCFAs in the TF of lambs is shown in Table 5. The concentrations of MOA in the DF were closely related to the EOA concentrations ([ρ] = 0.61, p = 0.001), but weaker correlations existed between MOA and MNA ([ρ] = 0.49, p = 0.016) and EOA and MNA ([ρ] = 0.45, p = 0.029). The concentrations of MOA and MNA ([ρ] = 0.29, p = 0.17) and EOA and MNA ([ρ] = 0.20, p = 0.34) had no significant correlation in the OF, although there was a tendency for correlation of the concentrations of MOA and EOA ([ρ] = 0.39, p = 0.06). The concentrations of MOA in the PF were closely related to the EOA concentrations ([ρ] = 0.70, p = 0.001) and the MNA concentrations ([ρ] = 0.66, p = 0.001), but a weaker correlation existed between EOA and MNA ([ρ] = 0.57, p = 0.004).

## DISCUSSION

### Animal growth performance and carcass characteristics

The final body weight and ADG were unaffected by dietary supplementation of *Allium mongolicum* Regel or its extracts. In contrast, Du et al [17] reported that dietary supplementation of *Allium mongolicum* Regel powder and its residue could influence the rumen microbiome and increase the ADG. This difference was probably because our experiment was conducted from April to July (vs July to October), and the average maximum temperature was 31°C (vs 24°C), and extreme temperatures reached 39°C in July. Slimen et al [25] argued that heat exposure could cause oxidative stress, which results from reactive oxygen species formation and can thus have an impact on weight gain. Similarly, treatment with *Allium sativum* essential oil and *Allium fistulosum* ethanolic and aqueous extracts did not affect body weight [26]. In the present study, the DMI was lower for the AFE than for the other groups, probably because the ethanol soluble *Allium mongolicum* Regel extract had a strong odor and flavor. Furthermore, several studies have reported that plant extracts can have a strong odor, flavor, and poor palatability, leading to a decrease in feed intake [3,27]. Li et al [28] reported that the FCR was significantly increased by *Allium mongolicum* Regel flavonoids acting as immunomodulators in *Channa argus*. In line with our results, dietary supplementation with the ethanol extract could significantly promote feed efficiency, indicating that the ethanol extract may contribute to the efficient utilization of feed even though the DMI decreased. Some studies have observed that the meat from pasture-fed lambs was darker than meat from stall-fed lambs and that subcutaneous dorsal fat was firmer in pasture-fed than stall-fed lambs, which was probably attributed to the lower level of unsaturated fatty acids due to a higher ruminal retention time and therefore greater ruminal biohydrogenation [29,30], which is taken into account by consumers. In this study, *Allium mongolicum* Regel and its extracts did not affect the DF firmness.

### Color of fat

Fat color is an important parameter for evaluating meat quality, and consumers around the world generally do not accept yellow fat [31]. In our study, *Allium mongolicum* Regel supplementation tended to make the PF and OF less yellow, and the redness and chroma in the OF were lower in AFE lambs than in CK lambs, as indicated by the lower b*, a*, and C* values, respectively, which might be related to the antioxidant activity of *Allium mongolicum* Regel, as speculated by Arnold [32], who reported that lipid oxidation and color development in ruminant meat is influenced by both fatty acid composition and the tissue concentration of antioxidant compounds. *Allium mongolicum* Regel is a type of perennial and xerophytic Liliaceous allium plant and is rich in flavonoids and polysaccharides, which have high antioxidant activity. Previous studies also reported that the *Allium mongolicum* Regel water extract and methanol extract, having higher phenolic contents (10.20 and 7.50 mg/g dry weight, respectively) and flavonoid contents (4.02 and 3.13 mg/g dry weight, respectively) than those of the powder, showed better antioxidant activity [33]. In this regard, the presence of antioxidant compounds in *Allium mongolicum* Regel may explain its antioxidant and radical scavenging activity. Therefore, the enrichment of antioxidants in the meat might have improved fat color in the present study. Compared to those of the AMR lambs, the PF and OF of the AFE lambs exhibited more yellowness, and more redness and chroma were observed in the OF of these lambs than in the OF of the AMR lambs. It is speculated that the *Allium mongolicum* Regel ethanol extract may have affected the fat color and deposition process of the pigments in fat, which may occur faster in internal fat (PF and OF) than in external fat (DF and TF) and would be in line with these differences [34]. Many studies have reported that dietary plant pigment (i.e., lycopene and carotenoids) supplementation can deposit into different tissues in humans and monogastric animals [35]. However, for sheep, there has been no specific study of the effect of dietary pigment exposure on the digestion, absorption, and metabolism of animal fat. Therefore, the effect of dietary *Allium mongolicum* Regel and its extracts on fat color should be further investigated. Nevertheless, in our study, supplementation with *Allium mongolicum* Regel and its extracts in lambs did not affect TF, DF, PF, or OF color compared to CK.

### Body fat three BCFAs concentrations

Flavor is one of the most essential sensory attributes of meat, which significantly affects the acceptance and purchasing decisions of consumers. Moreover, studies have shown that MOA and EOA are the main determinants of mutton odor and flavor [10]. It was also found that the flavor threshold levels of three BCFAs at pH 2 are increasing in the order EOA<MOA<MNA, as seen by Brennand et al [36]. The flavor threshold value is the lowest concentration range of compounds that can be determined by panelists and can be used to estimate the correlation between three BCFAs and characteristic odor and flavor. Accordingly, the MNA has a high flavor threshold, which most likely contributed only slightly to the characteristic odor and flavor. In this study, dietary supplementation with *Allium mongolicum* Regel and its extracts could decrease the MOA and EOA concentrations of the DF, OF, and PF in lambs but not those of the TF. This result was similar to the results obtained for flavonoids from *Allium mongolicum* Regel, all treated animals exhibited considerably decreased concentrations of MOA related to flavor in the *longissimus dorsi* muscle, which improved mutton flavor [15]. In summary, it can be predicted that *Allium mongolicum* Regel and its extracts have a positive effect on the reduction of the characteristic flavor and odor of lambs.

It is generally known that rumen microorganisms may isomerize unsaturated fatty acids after the degradation of triglycerides into BCFAs through biological hydrogenation. Moreover, some studies have suggested that feeding with *Allium mongolicum* Regel polysaccharides [14] and flavonoids [28] promotes the antioxidant status in juvenile northern snakehead fish. Therefore, we hypothesized that the antioxidant bioactive components in *Allium mongolicum* Regel and its extracts can block the biological hydrogenation of rumen microorganisms and reduce the production of BCFAs. Moreover, in ruminants, the degradation of valine and leucine amino acids provides raw materials for the synthesis of iso-acids, rather than the addition of methylmalonyl-CoA. We hypothesized that supplementation with *Allium mongolicum* Regel and its extracts decreased the proportion of iso-acids by altering the synthesis of iso-acids by ruminal microorganisms, resulting in three BCFAs lower in external (DF) or internal adipose tissues (OF and PF). Therefore, the effect of dietary *Allium mongolicum* Regel and its extracts on ruminal microorganisms should be further investigated.

In this study, as treatment with *Allium mongolicum* Regel and its extract did not significantly reduce the concentrations of BCFAs in the TF of small-tailed Han sheep, we hypothesized that the TF may present unique lipid metabolism traits in terms of regulating the concentrations of BCFAs in lambs. Alves et al [37] found high proportions of BCFAs in the fat TF of Damara rams, which have a unique, hanging fat tail, which acts as a fat deposit that can be mobilized under nutritional stress, among other harsh production conditions. Low-quality roughage diets contain a high proportion of fiber digesting bacteria, which are efficiently digested to produce high acetate levels and low propionate levels in the rumen. Therefore, to adapt to low-quality roughage diets, it is possible that the Damara breed also presents a lower threshold for propionate metabolism, thus, more propionate might be available to the TF, or regulation leading to elevated concentrations of BCFAs due to an eventual lower rate of hepatic metabolism may occur [37]. Small-tailed Han sheep also have the characteristics of coarse feeding tolerance and strong adaptability. Therefore, we speculated that the unique metabolism mechanism of the TF indicates that the metabolism of BCFAs is not regulated by diet in small-tailed Han sheep.

### Concentrations of three BCFAs in different adipose tissues

Overall, the concentrations of three BCFAs were significantly greater in external fat (TF) than in internal fat (OF and PF). For the external fat, the concentrations of three BCFAs were significantly higher in the TF than in the DF. For the internal fat, although there was no significant difference in the deposition of the three BCFAs between the OF and PF, the concentrations of the three BCFAs in the OF were numerically greater than those in the PF. This might be explained by the relative quantitative importance of *de novo* synthesis of fatty acids compared to the uptake of exogenous fatty acids and the unique metabolism mechanism among types of adipose tissue. This is the first time that the concentrations of three BCFAs in different types of adipose tissue and, possibly, elsewhere (TF, DF, OF, and PF) in small-tailed Han sheep were reported. The main reason for a few published researches on the concentrations of three BCFAs is that there are low concentrations of these BCFAs in the fat of lambs and require specialized procedures of analysis [8].

Chemical analysis of adipose tissues showed unusually high amounts of methyl-branched chain fatty acids, which contribute to the decrease in the melting point of the fat [8]. To the best of our knowledge, the melting point of noninternal fat was lower than that of internal fat. A direct feature of this was that the fat tissue was abnormally soft, which was more obvious in the TF than in the OF and PF. In other words, the melting point of the TF was lower than that of the internal fat (OF and PF), a concept supported by our observation of higher concentrations of BCFAs in the TF than in the OF and PF. Another study showed that sebaceous glands produce a significant proportion of BCFAs, and in some specialized glands, such as harderian, meibomian, and uropygial glands, BCFAs comprise most fatty acids produced by fatty acid synthetase. This may also be another reason why more BCFAs were deposited in the TF.

In agreement with Kaffarnik et al [8], MOA was observed at higher concentrations (23 to 88 μg/g) than that of EOA (13 to 26 μg/g) and MNA (2.9 to 18 μg/g) in the subcutaneous adipose tissue of three different breeds of sheep. Similarly, Watkins et al [22] found that MOA was the most abundant BCFA, with the median MOA concentration being almost two-fold greater than that of EOA and ten-fold greater than that of MNA. There is strong evidence that both EOA and MNA were correlated with MOA (*r*^2^ = 0.64 and 0.73, respectively, p<0.05), but EOA and MNA were not correlated (*r*^2^ = 0.18) in subcutaneous adipose tissue [10]. It can be seen that the results of this study contrast those reported by Santos et al [18], but it should be noted that the sampling location (posterior end of the loin vs over the *gluteus medius*) and correlation analysis methods (Spearman vs Pearson) were different. MNA is considered to play a less essential role than MOA in mutton aroma since its flavor threshold in water is over 30 times greater than that of MOA. Nevertheless, it is remarkable that subthreshold interactions of flavors contributed by BCFAs are possible.

## CONCLUSION

In general, dietary supplementation with the *Allium mongolicum* Regel ethanol extract could significantly promote feed efficiency, although dry matter intake decreased. Moreover, in our study, supplementation with *Allium mongolicum* Regel and its extract in lambs did not affect TF, DF, OF, or PF color. This study also demonstrated that *Allium mongolicum* Regel and its extracts could decrease the MOA and EOA concentrations of body fat in lambs, except for TF. The MOA was the most abundant of the BCFAs, followed by EOA and MNA, and the correlations of the three BCFAs were not uniform in the different types of adipose tissue.

*Allium mongolicum* Regel powder may affect slower than water-soluble or ethanol extract-soluble extract, however, its biological activity may be stronger than water-soluble or ethanol-soluble extract, because of the combined effect of many active components. On the other hand, the biological activity of *Allium mongolicum* Regel powder was not as good as expected as a result of interaction with food compounds. Three BCFAs are responsible for the characteristic flavor and odor compounds of lamb meat. Due to the methyl or ethyl branch, the carbon C-4 represents a stereogenic center with the possible presence of one or both enantiomers in the respective samples. In other words, 4-alkyl-branched fatty acids may occur in both *S*- and *R*-forms in body fat of lambs. Therefore, we should use the enantioselective gas chromatography to study the enantiomeric composition in future research.

## Figures and Tables

**Table 1:** Composition and analysis of experimental diets

Items	Numerical value
Ingredients (% dry matter basis)	
Chinese wildrye grass hay	32.00
Alfalfa hay particles	17.80
Corn	23.00
Wheat bran	2.87
Sunflower meal cake	16.92
Pea straw	2.45
Pomace	2.45
Dicalcium phosphate	0.31
Sodium chloride	0.34
Calcium carbonate	0.41
Sodium bicarbonate	0.33
Magnesia	0.12
Vitamin premix^1)^	1.00
Nutrient level (dry matter basis)	
Digestible energy^2)^ (MJ/kg)	16.90
Crude protein (g/kg)	170.20
Ether extract (g/kg)	63.00
Neutral detergent fiber (g/kg)	379.00
Acid detergent fiber (g/kg)	296.00
Calcium (g/kg)	13.90
Phosphorus (g/kg)	5.10

1) The mineral/vitamin premix contained the following: 25 mg of Fe (as ferrous sulfate), 29 mg of Zn (as zinc sulfate), 8 mg of Cu (as copper sulfate), 30 mg of Mn (as manganese), 0.04 mg of I (as potassium iodide), 0.1 mg of Co (as cobalt sulfate), 3,200 IU of vitamin A, 1,200 IU of vitamin D_3_, and 20 IU of vitamin E. The premix values are presented amount per kg of diet.

2) Digestible energy is a calculated value, whereas the other levels are measured values.

**Table 2:** Effect of dietary supplementation with *Allium mongolicum* Regel and its extracts on the growth performance and carcass characteristics of lambs

Items	Treatment^1)^				SEM	p-value^2)^

	CK	AMR	AWE	AFE		
Consumption amount of AMR (g)	0	11,250	11,250	11,250	-	-
Initial body weight (kg)	24.10	23.60	24.10	22.90	0.90	0.770
Final body weight (kg)	40.60	41.10	41.40	39.60	1.17	0.760
Dry matter intake (kg/d)	1.96^a^	1.99^a^	1.99^a^	1.83^b^	0.03	0.001
Average daily gain (g/d)	239.00	252.00	247.00	240.00	9.14	0.740
Feed conversion ratio	0.14^b^	0.14^b^	0.13^b^	0.15^a^	0.01	0.039
Hot carcass weight (kg)	21.90	21.10	21.20	21.00	0.44	0.420
Carcass yield (%)	53.00	51.70	53.70	52.70	0.89	0.450
DF thickness^3)^ (mm)	3.50	3.20	3.30	3.10	0.14	0.530
PF weight^4)^ (g)	244.00	213.00	221.00	211.00	8.21	0.670
DF firmness^5)^	4.00	3.30	3.50	3.20	0.13	0.590

SEM, standard error of the mean.

1) CK, a basal diet without additives; AMR, a basal diet supplemented with 10 g/lamb/d dried leaf powder of *Allium mongolicum* Regel; AWE, a basal diet supplemented with 3.4 g/lamb/d water extract of dried *Allium mongolicum* Regel leaf powder; AFE, a basal diet supplemented with 2.8 g/lamb/d ethanol extract of dried *Allium mongolicum* Regel leaf powder.

2) a,b Means within a row with different superscripts are different at p<0.05, whereas the differences were considered to be a statistical trend when 0.05<p<0.10. The number of observations for each mean value was fifteen (n = 15), except for hot carcass weight and carcass yield (n = 6).

3) DF thickness, dorsal subcutaneous adipose tissue thickness.

4) PF weight, perirenal adipose tissue weight.

5) DF firmness, dorsal subcutaneous adipose tissue firmness. DF firmness was measured using a scale from 3 (oily) to 15 (very hard).

**Table 3:** Effect of dietary supplementation with *Allium mongolicum* Regel and its extracts on the color of the various tissues in lambs

Item	Treatment^1)^				SEM	p-value^2)^

	CK	AMR	AWE	AFE		
Perirenal adipose tissue						
Lightness (L*)	81.45	81.49	81.96	80.76	0.89	0.820
Redness (a*)	6.45	5.81	5.41	6.61	0.66	0.540
Yellowness (b*)	6.13^ab^	5.96^b^	6.50^ab^	7.19^a^	0.46	0.096
Chroma (C*)	9.07	8.64	8.45	9.95	0.54	0.220
Hue angle (H*)	88.92	88.97	89.19	89.05	0.11	0.340
Tail adipose tissue						
Lightness (L*)	82.98	83.27	84.81	84.74	0.85	0.300
Redness (a*)	3.45	3.38	3.55	2.90	0.46	0.750
Yellowness (b*)	7.35	6.16	6.81	6.81	0.39	0.220
Chroma (C*)	8.22	7.15	7.87	7.48	0.44	0.350
Hue angle (H*)	89.52	89.42	89.47	89.48	0.09	0.860
Dorsal subcutaneous adipose tissue						
Lightness (L*)	85.95	84.33	84.53	84.70	0.79	0.470
Redness (a*)	4.64	4.40	5.25	4.37	0.65	0.760
Yellowness (b*)	7.68	6.94	6.25	7.46	0.46	0.140
Chroma (C*)	9.12	8.39	8.40	8.77	0.60	0.800
Hue angle (H*)	89.38	89.35	89.17	88.92	0.25	0.560
Omental adipose tissue						
Lightness (L*)	83.45	83.06	83.17	82.20	0.81	0.730
Redness (a*)	4.21^ab^	3.50^b^	3.96^b^	5.80^a^	0.59	0.044
Yellowness (b*)	4.99^ab^	4.23^b^	5.25^ab^	5.54^a^	0.37	0.087
Chroma (C*)	6.68^ab^	5.56^b^	6.86^ab^	8.11^a^	0.55	0.021
Hue angle (H*)	89.15	89.11	89.07	88.93	0.12	0.570

SEM, standard error of the mean.

1) CK, a basal diet without additives; AMR, a basal diet supplemented with 10 g/lamb/d dried *Allium mongolicum* Regel leaf powder; AWE, a basal diet supplemented with 3.4 g/lamb/d water extract of dried *Allium mongolicum* Regel leaf powder; AFE, a basal diet supplemented with 2.8 g/lamb/d ethanol extract of dried *Allium mongolicum* Regel leaf powder.

2) a,b Means within a row with different superscripts are significantly different at p<0.05, whereas the differences were considered to be a statistical trend when 0.05<p<0.10. The number of observations for each mean value was six (n = 6).

**Table 4:** Effect of dietary supplementation with *Allium mongolicum* Regel and its extracts on the concentrations of three branched-chain fatty acids (μg/g) in the various tissues in lambs

Item	Treatment^1)^				SEM	p-value^2)^

	CK	AMR	AWE	AFE		
Perirenal adipose tissue						
4-methyloctanoic acid	10.72^a^	4.05^b^	3.92^b^	2.31^c^	0.34	<0.001
4-methylnonanoic acid	1.14^a^	0.50^b^	0.48^b^	0.33^b^	0.21	0.044
4-ethyloctanoic acid	5.40^a^	1.99^b^	1.97^b^	1.05^b^	0.46	<0.001
Tail adipose tissue						
4-methyloctanoic acid	21.92	21.79	21.56	18.67	2.96	0.840
4-methylnonanoic acid	2.22	2.18	2.13	1.93	0.41	0.960
4-ethyloctanoic acid	11.47	11.28	11.68	10.25	1.49	0.610
Dorsal subcutaneous adipose tissue						
4-methyloctanoic acid	16.44^a^	7.29^b^	7.04^b^	6.27^b^	0.86	<0.001
4-methylnonanoic acid	1.64	0.72	0.70	0.64	0.36	0.180
4-ethyloctanoic acid	9.21^a^	3.57^b^	3.42^b^	3.15^b^	0.41	<0.001
Omental adipose tissue						
4-methyloctanoic acid	13.64^a^	4.91^b^	4.56^b^	4.08^b^	0.89	<0.001
4-methylnonanoic acid	1.16^a^	0.66^b^	0.61^b^	0.56^b^	0.16	0.062
4-ethyloctanoic acid	7.03^a^	2.66^b^	2.49^b^	2.16^b^	0.69	0.002

SEM, standard error of the mean.

1) CK, a basal diet without additives; AMR, a basal diet supplemented with 10 g/lamb/d dried *Allium mongolicum* Regel leaf powder; AWE, a basal diet supplemented with 3.4 g/lamb/d water extract of dried *Allium mongolicum* Regel leaf powder; AFE, a basal diet supplemented with 2.8 g/lamb/d ethanol extract of dried *Allium mongolicum* Regel leaf powder.

2) a,b Means within a row with different superscripts are significantly different at p<0.05, whereas the differences were considered to be a statistical trend when 0.05<p<0.10. The number of observations for each mean value was six (n = 6).

**Table 5:** Correlation analysis of three branched-chain fatty acids in the various tissues in lambs

Item	4-methyloctanoic acid		4-methylnonanoic acid		4-ethyloctanoic acid	
		
	ρ^1)^	p-value^2)^	ρ	p-value	ρ	p-value
Perirenal adipose tissue						
4-methyloctanoic acid	-	-	0.66	0.001	0.70	0.001
4-methylnonanoic acid	0.66	0.001	-	-	0.57	0.004
4-ethyloctanoic acid	0.70	0.001	0.57	0.004	-	-
Tail adipose tissue						
4-methyloctanoic acid	-	-	−0.04	0.85	−0.15	0.48
4-methylnonanoic acid	−0.04	0.85	-	-	0.13	0.55
4-ethyloctanoic acid	−0.15	0.48	0.13	0.55	-	-
Dorsal subcutaneous adipose tissue						
4-methyloctanoic acid	-	-	0.49	0.016	0.61	0.001
4-methylnonanoic acid	0.49	0.016	-	-	0.45	0.029
4-ethyloctanoic acid	0.61	0.001	0.45	0.029	-	-
Omental adipose tissue						
4-methyloctanoic acid	-	-	0.29	0.17	0.39	0.06
4-methylnonanoic acid	0.29	0.17	-	-	0.20	0.34
4-ethyloctanoic acid	0.39	0.06	0.20	0.34	-	-

1) ρ, Spearman’s rank correlation coefficients analysis.

2) The data means were considered significantly different at p-value<0.05, and tendencies were considered at 0.05<p-value<0.10. The number of observations for each mean value was twenty-four (n = 24).
